# Supplementary material for: Towards unified AI-driven fracture mechanics: the extended deep energy method (XDEM)
Source: Nat Commun. 2026 Aug 15;17:8492. doi: 10.1038/s41467-026-76748-1 (PMC13484471; doi:10.1038/s41467-026-76748-1)
Supplement: Supplementary file 3 [file 41467_2026_76748_MOESM3_ESM.zip › pic/Results/Stress_intensity_factor/Cross/comparison_xdem_fem/angle_75/fem_dem_error_uv_3x2.pdf]

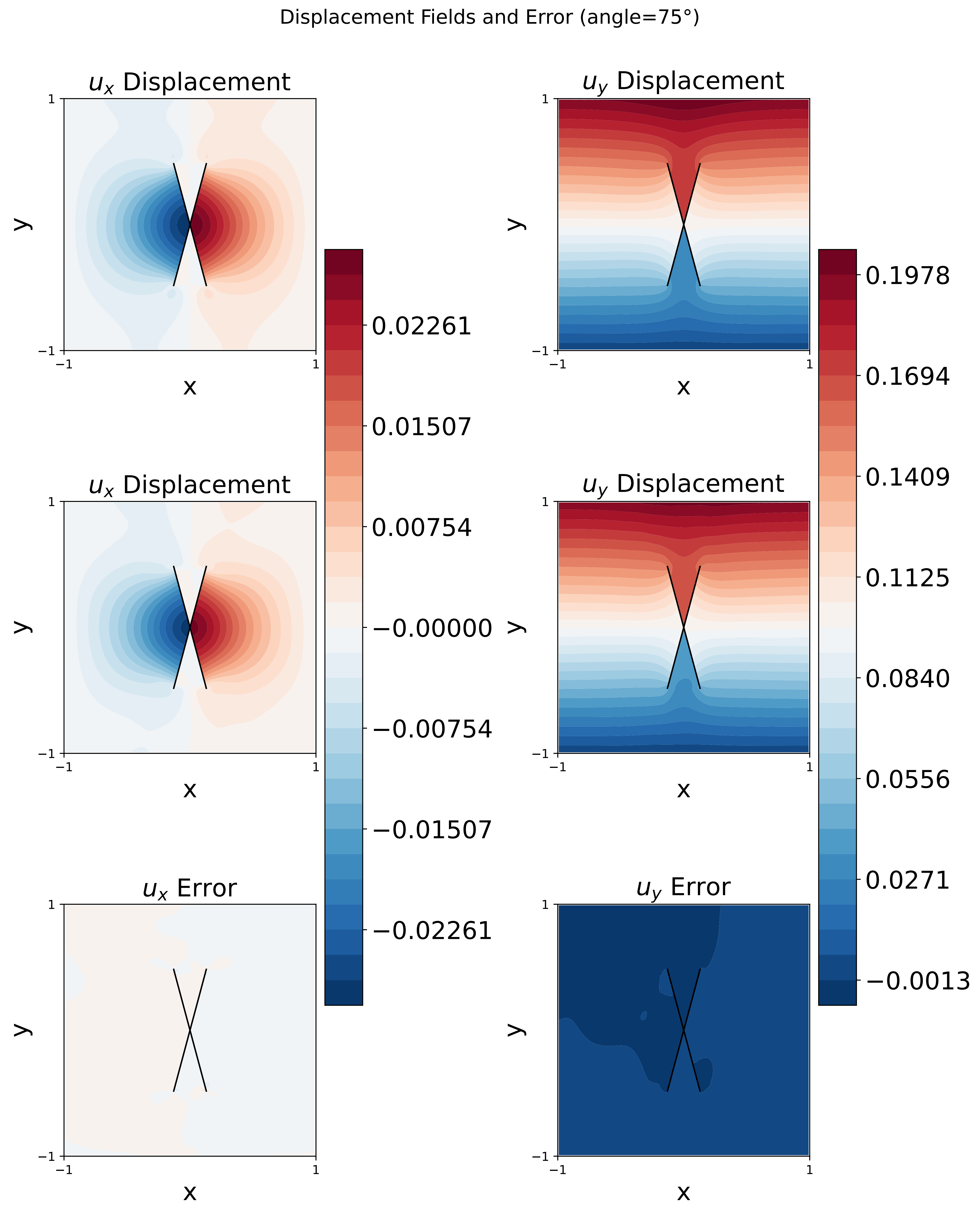
Displacement Fields and Error (angle=75°)
u_x Displacement
u_y Displacement
u_x Displacement
u_y Displacement
u_x Error
u_y Error
x
y
0.02261
0.01507
0.00754
−0.00000
−0.00754
−0.01507
−0.02261
0.1978
0.1694
0.1409
0.1125
0.0840
0.0556
0.0271
−0.0013
